# Case Report: Persistent toxic reactions in a toddler with a negative blood cantharidin toxicology test

**DOI:** 10.3389/fped.2025.1546669

**Published:** 2025-02-27

**Authors:** Zhuyan Duan, Yanning Qu, Rui Tang, Mengyi Sheng, Liangliang Wang, Jiao Li, Si Zheng, Linying Guo

**Affiliations:** ^1^ Emergency Department, Children's Hospital Affiliated to Capital Institute Pediatrics, Beijing, China; ^2^ Institute of Medical Information, Chinese Academy of Medical Sciences and Peking Union Medical College, Beijing, China

**Keywords:** cantharidin, poisoning, toxic reactions, persistent, toxicology test

## Abstract

Cantharidin is a potent natural toxin and has been used in traditional Chinese medicine to treat cancers and various ailments such as rabies or psoriasis. However, improper use of cantharidin can easily lead to poisoning. Despite there is an increasing amount of literature on the toxic mechanisms of cantharidin, there is still limited knowledge about the disease progression, toxic reactions and toxic blood concentrations in children with cantharidin poisoning. Caregivers of children suffering from cantharidin poisoning might fail to provide an accurate exposure dose when the children exhibit symptoms of intoxication. Moreover, the detection results on routine blood drug screens may be negative, thus delaying the clinical diagnosis and treatment. This study describes a case of cantharidin poisoning in a 2-year-old boy who ingested a traditional Chinese herbal remedy for dog bites. The toddler exhibited severe symptoms related with digestive and urinary systems. Though cantharidin was no longer detectable in the blood 20 h after admission, the poisoning symptoms persisted for approximately 5 days, and ultrasound showed that there was still sediment in the bladder two weeks after discharge. This case highlights the need for clinicians to consider the widespread tissue distribution of cantharidin, which may lead to prolonged toxic reactions.

## Introduction

Cantharidin is a bicyclic terpene toxin secreted by beetles of the Meloidae family. It has extremely strong biological activity, particularly in anticancer and anti-fibrosis applications. For example, cantharidin can inhibit intracellular phosphatase activity, leading to cytoskeleton disintegration and apoptosis (1, 2), or induce oxidative stress and inflammatory responses, directly causing cell necrosis (3).

However, cantharidin also exhibits broad tissue toxicity, with significant effects on the digestive tract and urinary system (4, 5). Poisoning patients may experience severe abdominal pain, vomiting, and bloody stools, and in some cases, it can lead to serious adverse reactions or even death.

Due to its high toxicity, modern medicine has gradually reduced the use of cantharidin. However, in China, cantharidin has been an important component of traditional Chinese medicine for thousands of years and continues to be used in folk remedies to treat conditions such as rabies and skin diseases (6). Because the therapeutic dose of cantharidin is very close to its toxic dose, poisoning or death can occasionally occur due to incorrect use.

Cantharidin poisoning in children often results from rabies prevention or accidental ingestion of blister beetles. Despite its significance, knowledge about persistent toxic reactions and toxicant metabolism in children with cantharidin poisoning remains limited, and most children affected by cantharidin poisoning do not undergo toxicological testing. Early recognition and timely treatment are crucial for improving outcomes.

With the deepening understanding of the toxicity of cantharidin, cases of cantharidin poisoning in children have significantly decreased, though occasional incidents still occur and can lead to severe consequences. Therefore, it is crucial to promptly summarize the clinical manifestations and practical experience related to cantharidin poisoning in pediatric patients. This article discusses clinical presentation, diagnostic methods, and treatment strategies of a toddler with cantharidin poisoning. By combining a case analysis with a literature review on cantharidin poisoning in children, the study aimed to summarize the current research progress and future directions, providing guidance for pediatric clinical practice.

## Case presentation

A 2-year-2-month-old boy admitted to the emergency department after vomited three times and experienced one hematuria within one day. The toddler comes from a rural part of Beijing. Seventeen days before admission, the toddler was bitten by a dog. Eight hours before admission, he was given a dose of traditional Chinese herbal remedy containing dried bodies of cantharides by his caregivers, for rabies prevention. After taking the folk remedy, he immediately induced vomiting symptoms. Worried about the ineffectiveness of the drug, the caregivers gave the same dose of folk remedy to the toddler again. However, half an hour later, the toddler vomited again, with invisible drug and food remnants in the vomitus. Later, the child began to have intermittent crying, accompanied with abdominal discomfort and frequent retching. When the toddler vomited again, the caregivers paused feeding and gave only small amounts of water to him. One hour before admission, the toddler had hematuria, with visible blood clots in the urine. At this time, there was no fever, no diarrhea, but the toddler appeared lethargic and had poor appetite. These prompted the caregivers to seek emergency medical attention. They arrived at the emergency department by private car.

Upon arrival at the emergency department of children's hospital, Primary physical examination results were as follows: Body temperature: 36.5°C, heart rate: 110 beats/min, respiratory rate: 20 breaths/min, blood pressure: 112/71 mmHg. Further assessment showed that the toddler was conscious but had a sluggish response, the breathing is stable, the abdomen is soft, without rebound tenderness and muscle rigidity. The liver and spleen were not palpable, bowel sounds were weak, and no abnormalities were noted in the heart, lungs, or nervous system examinations.

**Figure 1:**
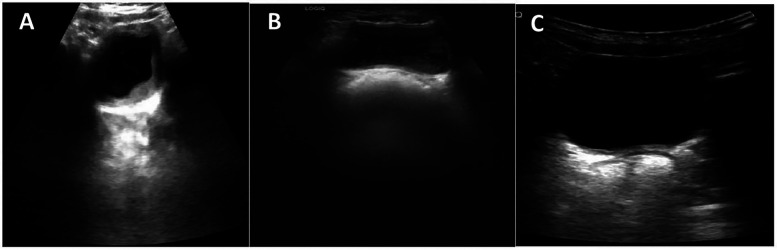
Ultrasonographic images of the bladder at different time points. **(A)** Bladder ultrasound on the first day of admission, showing sediment in the bladder. **(B)** Follow-up bladder ultrasound performed two weeks post-discharge, revealing there's still visible bladder sediment. **(C)** Follow-up bladder ultrasound one month post-discharge, showing the bladder sediment disappeared.

Laboratory tests showed elevated white blood cell count (16.63 × 10^9^/L) and neutrophils proportion (83.8%). The C-reactive protein (0.79 mg/L), hemoglobin (127 g/L) and platelets (413 × 10^9^/L) were normal, indicating that the toddler does not have anemia or thrombocytopenia. The urine was turbid and brown, with a pH of 6.5 and protein level of 6.0 g/L (3+), the microscopic examination showed 8–10 white blood cells/HP and numerous red blood cells per high-power field. Other indicators such as liver enzymes, cardiac enzymes, creatinine, blood urea nitrogen, electrolytes, and coagulation tests were all within normal ranges. However, the abdominal ultrasound revealed sediment in the bladder (Figure 1A). These results, along with the toddler's acute gastrointestinal symptoms and the caregivers' description that the folk remedy might contain dry bodies of cantharides, led to the diagnosis of cantharidin poisoning.

The medical providers suggested doing a blood test for cantharidin concentration (using gas chromatography-mass spectrometry) to help confirm the diagnosis, but his caregivers refused. Then, the toddler received treatment at the emergency observation ward. Briefly, the toddler was treated with intravenous ceftriaxone (50 mg/kg) for anti-infection, intravenous vitamin B6 (25 mg) for antiemetic treatment, intravenous vitamin K1 (5 mg) and ethamsylate (0.25 g) for hemostasis. Meanwhile, the toddler was given sodium bicarbonate infusion for urine alkalinization (along with oral administration), with a total fluid intake of 2,000–3,000 ml/m^2^. About 20 h later, the caregivers agreed to do blood cantharidin concentration testing for the toddler and the test yielded a negative result. It should be noted that the qualitative detection limit for cantharidin blood concentration using gas chromatography-mass spectrometry (GC-MS) is 1 ng/ml, and the quantitative detection limit is 3 ng/ml. Although the cantharidin concentration in the blood was not detectable in this case (below the qualitative detection limit), this does not completely rule out the possibility of poisoning in the toddler, as the toxin may have been metabolized or distributed to other tissues that are not reflected in the blood. At the same time, the toxicological tests for the folk remedy showed it contains cantharidin at a concentration of 1 µg/mg. Combined with the ingested dosage of the toddler, the medical providers inferred that the toddler has consumed approximately 300 µg of cantharidin. A diagnosis of cantharidin poisoning was clearly made for the toddler.

On the third day of admission, the toddler's urine color returned to normal. Further microscopic examination of the urine revealed no red blood cells. Also, no abnormalities were observed from blood tests, biochemical tests and coagulation tests. Although the acute symptoms seemed under control, close monitoring for potential delayed toxic reactions was still needed for the toddler.

On the fourth day of admission, however, the toddler started vomiting again. He vomited five times, with fever, and the maximum temperature is 39°C. A high white blood cell was observed (20 × 10^9^/L), the proportion of neutrophils was 78.6%, and the CRP increased to 1.55 mg/L. These findings suggest that the toddler may have developed acute gastroenteritis. Therefore, intravenous ceftriaxone (50 mg/kg) and supportive fluid therapy were continued. At this time, it was still necessary to distinguish between the delayed toxic reactions of cantharidin poisoning and a new infection event, and the toddler's overall health condition still required continuous monitoring.

On the fifth day of admission, the toddler's temperature returned to baseline, the vomiting relieved. The toddler's condition gradually stabilized, all indicators returned to normal, and he was discharged on the sixth day of hospitalization. Urinary ultrasound examination at the two-week post-discharge follow-up revealed that there was still visible bladder sediment (Figure 1B), which had disappeared by the one-month post-discharge (Figure 1C). All other test results had returned to normal at the 4-month follow-up.

## Discussion

This case report is unique in that the toxic effects in the child persisted for a prolonged period even after the blood toxicology results were negative. Currently, there is no specific antidote for cantharidin poisoning, and treatment is primarily supportive, aimed at alleviating symptoms, promoting toxin elimination, and preventing complications (7). Pediatricians should be aware of the key signs and symptoms of cantharidin poisoning in children and develop appropriate diagnostic tests and clinical management strategies. For example, in the early stages, gastric lavage and the use of activated charcoal can help reduce the absorption of cantharidin in patients. However, gastric lavage must be performed within 1–2 h of toxin ingestion, and only if the patient is conscious and does not have swallowing difficulties. Adequate fluid replacement is generally required to maintain blood volume and promote toxin excretion. For patients at risk of acute kidney injury, renal function should be closely monitored, and protective measures should be taken, such as urine alkalization, the use of diuretics (e.g., furosemide), and avoiding nephrotoxic drugs. Monitoring and correction of electrolyte imbalances, such as supplementation of sodium, potassium, and calcium, are essential. For patients with severe acute kidney injury, hemodialysis or peritoneal dialysis may be required to maintain renal function.

In this case, the possibility of cantharidin poisoning was promptly recognized based on exposure to folk remedies and the clinical presentation. Laboratory tests revealed a significant number of red blood cells and proteinuria, but no significant abnormalities in the cardiac, hepatic, or renal function, which provided crucial clues for evaluating potential multi-system involvement. Upon admission, the child received aggressive fluid resuscitation, alkalinization, anti-infection, antiemetic, and hemostatic treatments, which quickly relieved the symptoms and normalized laboratory values. However, on the fourth day, the child experienced vomiting and fever again, suggesting the need for close monitoring of complications, such as acute gastroenteritis.

Data of cantharidin poisoning in 26 children from publications (8–22) (Table 1) indicated that the peak incidence of cases occurred in the 1960s and 1990s. In recent years, with increased public awareness, the incidence of poisoning has significantly declined. Another reason for the recent decline in reports may be that physicians see no need to report yet another case. The only way to accurately assess the incidence of poisoning would be through a registry or admission records. In China, pediatric poisoning is mostly associated with rabies prevention, whereas in other countries, it is often caused by accidental ingestion of blister beetles. Besides, urinary symptoms (hematuria, proteinuria, and renal impairment), gastrointestinal symptoms (vomiting, diarrhea, hematochezia), neurological symptoms and local mucosal damage were present in 91.6%, 75%, 50% and 25% of cases, respectively. Treatment in majority of these reports was primarily supportive. In some cases, antibiotics were used to prevent or control infections, and hormones were administered in certain instances. Though some patients showed elevated creatinine and blood urea nitrogen levels, none developed severe acute renal failure requiring blood purification. Although most patients showed positive recovery, with an average hospital stay of 9.3 days, the mortality rate was 12.5%, suggesting that cantharidin poisoning remains a serious and concerning issue, as poisoning or death can still occur, and not only in rare cases. None of the previous studies reported the blood cantharidin concentration testing results in children affected by cantharidin poisoning. We still have little knowledge about the association between cantharidin doses and toxic reactions in children.

**Table 1:** Summary of children with acute cantharidin poisoning.

Year	Age and gender	Location	Clinical manifestation					Outcome
			Gastrointestinal	Urinary	Circulatory	Localized corrosion	Nervous	
1960 (8)	22 m, M	African	−	+	−	+	−	Discharge after 9 days
1960 (9)	17y, M	Philadelphia, USA	+	+	−	+	−	Discharge after 5 days
1964 (10)	12y, M	China	+	+	−	−	−	Discharge after 18 days
1964 (11)	6y, M	Dalian, China	+	+	−	−	−	Discharge after 3 days
1966 (12)	6y, M	China	−	−	−	+	−	Death after 12h
1966 (12)	4y, M	China	+	+	−	+	+	Discharge after 13 days
1966 (12)	9y, M	Jiangxi, China	+	+	−	−	+	Discharge after 13 days
1967 (13)	10 m, F	Missouri, USA	+	+	−	−	−	Discharge after 11 days
1987 (14)	4y, M	Jiangxi, China	+	+	−	−	−	Discharge after several days
1991 (15)	7y, M	China	+	+	−	−	+	Discharge after 7 days
1991 (15)	11y, M	China	−	+	−	−	−	Discharge after 5 days
1991 (15)	4y, M	China	+	+	−	−	+	Discharge after 12 days
1994 (16)	6y, M	Guiyang, China	+	+	−	−	−	Discharge after 4 days
1994 (16)	11y, M	Guiyang, China	+	+	−	−	−	Discharge after 3 days
1994 (16)	7y, F	Guiyang, China	+	+	−	−	+	Discharge after 28 days
1996 (17)	5y, M	Oman	+	+				Discharge after 7 days
1996 (17)	2y, F	Oman	+	+				Discharge after 7 days
2000 (18)	4y, F	African	+	+	−	−	+	Discharge after 9 days
2000 (18)	7y, M	African	+	−	−	−	+	Death
2003 (19)	3y, M	Shandong, China	−	+	−	−	−	Discharge after 12 days
2003 (19)	2y, M	Shandong, China	−	+	−	+	+	Death
2003 (19)	11y, M	Shandong, China	−	+	−	−	−	Discharge after 5 days
2010 (20)	8 m, M	Saudi Arabia	+	+	−	−	+	Discharge after several days
2010 (20)	11 m, F	Saudi Arabia	+	+	−	−	+	Discharge after 6 days
2014 (21)	2 m, F	Saudi Arabia	+	+	+	+	+	Discharge after 6 days
2018 (22)	18 m, F	Saudi Arabia	+	+	−	−	+	Discharge after 7 days

m, months; y, years; M, male; F, female; +, positive manifestation; −, negative manifestation.

Although cantharidin is used in certain therapeutic doses in traditional Chinese medicine, its safety in pediatric patients has not been fully established. The therapeutic dose of cantharidin is very close to its toxic dose, and even slight fluctuations in dosage may lead to severe toxic reactions. Particularly in children, whose organs are still developing, body weight is relatively low, and metabolic functions are weaker, the sensitivity to toxins is significantly higher than in adults. Previous literature reports indicate that the toxic dose of cantharidin typically ranges from 10 to 60 mg, with severe cases being fatal at an intake of 0.5–1 mg/kg (23). However, in our case, the toddler ingested approximately 300 µg of cantharidin (the actual intake may be lower due to vomiting), it still caused severe poisoning symptoms. Therefore, whether there is a “safe dose” of cantharidin for children remains unclear and requires cautious consideration. Negative blood cantharidin concentration result was acquired after 20 h of ingestion, but persistent poisoning symptoms had presented for about five days. To the best of our knowledge, only one pharmacokinetic study of cantharidin has been reported (24), where beagle dogs injected intravenously with 34 µg/kg of cantharidin showed a *t*_1/2_ of 0.69 ± 0.03 h and a v_ss_ of 0.24 ± 0.02 L/kg. This indicates that although cantharidin is metabolized relatively quickly, it is widely distributed in the body, primarily causing sustained toxicity in the kidneys and liver (25). Therefore, we suggested that clinicians should be aware of the potential ongoing toxic damage for pediatrics, even when the blood level was negative.

## Conclusion

This study reports a case of cantharidin poisoning in a 2-year-old child, marking the first time that blood cantharidin concentration testing was performed in a pediatric patient. Despite the negative result, the child exhibited persistent toxic symptoms, likely due to cantharidin's large volume of distribution. After a series of treatments, the child had a favorable outcome, highlighting the importance of considering ongoing toxicity even in the absence of detectable cantharidin concentration in blood toxicology testing in clinical practice.

## Data Availability

The raw data supporting the conclusions of this article will be made available by the authors, without undue reservation.
